# A Comparative Study of Item Response Theory Models for Mixed Discrete-Continuous Responses

**DOI:** 10.3390/jintelligence12030026

**Published:** 2024-02-25

**Authors:** Cengiz Zopluoglu, J. R. Lockwood

**Affiliations:** 1 College of Education, University of Oregon, Eugene, OR 97403, USA; 2 Duolingo, Inc., Pittsburgh, PA 15206, USA; jr@duolingo.com

**Keywords:** item response theory, bounded continuous data, continuous response model, dictation task, language assessment, natural language processing, zero-and-one inflated data

## Abstract

Language proficiency assessments are pivotal in educational and professional decision-making. With the integration of AI-driven technologies, these assessments can more frequently use item types, such as dictation tasks, producing response features with a mixture of discrete and continuous distributions. This study evaluates novel measurement models tailored to these unique response features. Specifically, we evaluated the performance of the zero-and-one-inflated extensions of the Beta, Simplex, and Samejima’s Continuous item response models and incorporated collateral information into the estimation using latent regression. Our findings highlight that while all models provided highly correlated results regarding item and person parameters, the Beta item response model showcased superior out-of-sample predictive accuracy. However, a significant challenge was the absence of established benchmarks for evaluating model and item fit for these novel item response models. There is a need for further research to establish benchmarks for evaluating the fit of these innovative models to ensure their reliability and validity in real-world applications.

## 1. Introduction

In educational and psychological assessments, continuous response features frequently arise either as an inherent characteristic of an assessment, such as in reading fluency measures (e.g., DIBELS, University of Oregon 2018–2020) or continuous rating scales (Bejar 1977; Brumfitt and Sheeran 1999) or as a consequence of summed dichotomous and polytomous item scores, such as in C-tests (Raatz and Klein-Braley 1981) or Maze assessments (Guthrie et al. 1974). In addition, the rapid evolution of machine learning and AI-driven technologies (Bommasani et al. 2021) offers new opportunities for more frequent and innovative use of constructed-response items from which continuous features can be derived. These features can be used as indicators of person-level proficiency traits and can yield inferences about these traits when combined with appropriate theoretical and measurement models.

In practice, it is common for “continuous” features to have observed distributions that are best described by a mixture of discrete and continuous distributions. For instance, when a continuous feature has a bounded distribution (e.g., between 0 and 1), it may have a nontrivial probability of being 0 and/or 1 when computed from a sample of responses from some target population. An example that we consider in this study arises from the use of edit distance (Levenshtein 1965) to evaluate the accuracy of a response to a dictation task, in which a test taker is asked to listen to a target sentence in English and then type the sentence in English (Buck 2001; Cardwell et al. 2023). If the typed response matches the target sentence, one could assign a grade of 1. Lower grades can be assigned as the typed response deviates more from the target sentence with respect to some edit distance. A grade of 0 can be assigned to responses for which the edit distance exceeds the length of the target sentence. Depending on the complexity of the target sentence and the distribution of English proficiency in the target population of test takers, the distribution of this grade may be best described by a mixture of discrete probability masses at 0 and 1 and a continuous distribution on the open unit interval (0, 1).

Building on the diverse applications of continuous response features in educational and psychological assessments, the psychometric literature has witnessed a significant evolution of Item Response Theory (IRT) models proposed for continuous response features. The pioneering works by Samejima (1973), who presented a Continuous Response Model as a special case of the Graded Response Model, and Müller’s extension (Müller 1987) of the Rating Scale Model, along with a simple linear response model by Mellenbergh (1994), laid the groundwork for subsequent models that catered to more complex data structures. These studies emphasized the need for models that could handle ceiling and floor effects inherent in continuous data. The exploration of bounded continuous response features, such as those ranging from 0 to 1, gained momentum with the contributions of Noel and Dauvier (2007), who presented a Beta Item Response Model. This model addresses the complexities and nuances of response distributions within bounded continuous data. This model incorporates an interpolation process as the response mechanism, leading to a beta distribution for responses. Therefore, unlike early IRT models, it effectively captures the asymmetric nature of such data, thereby enriching the theoretical and practical applications of IRT in bounded continuous data scenarios. Recent contributions have expanded the scope of continuous response modeling in IRT. Flores et al. (2020) introduced a model leveraging the simplex distribution to model response times within a bounded framework. This model significantly advances handling response features that exhibit continuous and discrete characteristics, particularly in time-limited assessment scenarios.

While Item Response Theory (IRT) models for bounded continuous features are well-studied, Molenaar et al. (2022) highlighted a significant limitation in these models, mainly when dealing with data concentrated at the boundaries. For instance, significant clustering at these limits may occur in assessments where responses are naturally bounded between 0 and 1. This clustering poses challenges for traditional IRT models, which typically do not account for such high concentrations at the boundary values. Molenaar et al. (2022) demonstrated that even a small proportion of responses at these boundaries could significantly impact parameter estimates, leading to potential biases in the interpretation of data. They proposed a set of new IRT models that better accommodate these boundary concentrations, providing more accurate and reliable analysis in such scenarios. These models are suitable for features with distributions having a mixture of discrete point masses and continuous values on a bounded interval. As these and other novel IRT models emerge, empirical evaluations of their performance with real data from diverse contexts are essential to the research and practitioner communities to improve measurement science.

In this study, we evaluate the performance of three novel IRT models, zero-and-one-inflated extensions of the Beta, Simplex, and Samejima’s Continuous IRT models proposed by Molenaar et al. (2022) by (a) using a dataset characterized by its extreme sparsity, (b) supplementing these models with a latent regression component, and (c) conducting model comparisons using cross-validation and posterior predictive checks. The dataset used in this study comes from a high-stakes English language proficiency assessment. It exhibits a substantial proportion of “1” grades and a smaller occurrence of “0” grades, providing a challenging yet ideal scenario for examining models designed to handle a mixture of discrete and continuous data. To address the sparse nature of our dataset, we incorporate latent regression, using standardized writing and speaking scores as auxiliary variables within the IRT framework to improve the accuracy and precision of parameter estimation. This integration is directly in line with established research suggesting that including auxiliary information leads to more accurate estimates. Moreover, we employ cross-validation and posterior predictive checks as a means of model evaluation.

## 2. Materials and Methods

### 2.1. Data

We analyzed responses from a sample of the Duolingo English Test test takers, a computerized-adaptive assessment of English language proficiency designed to support high-stakes decisions in English-medium settings (Cardwell et al. 2023). The sample consists of 295,157 test sessions from 222,568 unique test takers (some repeat the assessment). All sessions in the sample occurred before September 2022. Test takers in the sample represent more than 200 unique countries (India = 22%, China = 18%, all others < 5%) and 146 native languages (Mandarin = 19%, Spanish = 9%, English = 9%, Telugu = 8%, all others < 5%). Approximately 47% of test takers are female, 39% percent report the intention to apply to undergraduate programs, and another 39% percent report the intention to apply to graduate programs.

As previously described, we focus on the test takers’ responses to dictation items. This task aims to evaluate the test taker’s capacity to identify individual words and retain them in memory for a sufficient amount of time to reproduce them with precision. Each test taker in the dataset has responses to between 4 and 7 dictation items, with the majority (91.6%) responding to 6 items. The first dictation item is randomly assigned for each test taker, whereas the remaining items are assigned adaptively based on the test taker’s performance on previous items. As a consequence of adaptive assignment, no two test takers in the sample have responses to the same set of dictation items, making the person–item linkage structure highly sparse. The dataset consists of 1,789,297 responses to 2738 dictation items.

The response of a given test taker to a given item is graded on the interval [0, 1] using a function of the character-based edit distance between the target sentence and what is typed by the test taker. The grades are defined so that a value of 1 indicates an essentially perfect rendering of the target sentence, whereas lower grades correspond to increasing discrepancies. A minimum grade of 0 occurs when the edit distance equals or exceeds the length of the target sentence. Approximately 47% of the item responses receive a grade of 1. Grades of 0 occur but are rare (0.03%). The remaining 53% of grades have a mean of 0.87 and a standard deviation of 0.125. The dataset also includes each test taker’s score on the speaking and writing portions of the assessment, which we use in some models as predictors of dictation proficiency via latent regression. Details on the tasks contributing to these speaking and writing scores and other technical details about the assessment can be found in Cardwell et al. (2023).

### 2.2. Zero-and-One-Inflated Item Response Models for Bounded Continuous Data

Molenaar et al. (2022) discuss some novel IRT models designed for bounded continuous data, addressing the limitations of traditional models when applied to data constrained within the closed interval [0, 1]. These novel models are particularly adept at handling situations where responses are clustered at the boundaries of the scale, a common occurrence in educational and psychological assessments. The Beta IRT model assumes a beta distribution for the response propensity, characterized by its flexibility in modeling various shapes of response distributions. It is suitable for data with natural boundaries, like percentages or proportions, common in psychological scales. Based on the S_B_ distribution, the Continuous Response Model is a special case within Samejima’s Graded Response Model framework. The Simplex IRT model utilizes the simplex distribution, which, while less common, offers an alternative modeling approach to bounded continuous data. This model is beneficial in contexts such as response time analysis, where the data is naturally bounded within a specific range. In this section, we provide a general introduction to the overall model structure for all these models. All three models operate under the same structure, and the only difference is the model-specific density function utilized when modeling the continuous part of the distribution. 

Let $X_{pi}$ denote the continuous bounded item scores such that $X_{pi\;}\epsilon \;\left[0,\;1\right]$ for the pth person, $p=\left\{1,\;\ldots ,\;P\right\}$, on the ith item, $i=\left\{1,\;\ldots ,\;I\right\}$. We can define a discrete variable $Z_{pi}$, representing three possible conditions as the following,

$$Z_{pi}=\left\{\begin{array}{cc} 0, & if\;X_{pi}=0\\ 1, & if\;0<X_{pi}<1\\ 2, & if\;X_{pi}=1 \end{array}\right.$$

A logistic Graded Response Model (Samejima 1969) can be written for modeling $Z_{pi}$ such that,

$$P\left(Z_{pi}=0|\theta_{p},\alpha_{i},\gamma_{0i}\right)=\frac{1}{1+e^{\alpha_{i}\theta_{p}-\gamma_{0i}}}$$

$$P\left(Z_{pi}=1|\theta_{p},\alpha_{i},\gamma_{0i},\gamma_{1i}\right)=\frac{1}{1+e^{\alpha_{i}\theta_{p}-\gamma_{1i}}}-\frac{1}{1+e^{\alpha_{i}\theta_{p}-\gamma_{0i}}}$$

$$P\left(Z_{pi}=2|\theta_{p},\alpha_{i},\gamma_{1i}\right)=\frac{e^{\alpha_{i}\theta_{p}-\gamma_{1i}}}{1+e^{\alpha_{i}\theta_{p}-\gamma_{1i}}}$$

where $\theta_{p}\in R$ is a latent person parameter, $\alpha_{i}\;\in R^{+}$ is an item discrimination parameter, and $\gamma_{0i}\in R$ and $\gamma_{1i}\in R$ are category threshold parameters satisfying $\gamma_{0i}<\gamma_{1i}$.

Then, the joint conditional density for the model, which is denoted by $k\left(.\right)$, can be written as the following:

$$k\left(X_{pi}|\theta_{p},\alpha_{i},\gamma_{0i}\right)=P\left(Z_{pi}=0|\theta_{p},\alpha_{i},\gamma_{0i}\right)$$

$$k\left(X_{pi}|\theta_{p},\alpha_{i},\gamma_{0i},\gamma_{1i},\beta_{i},\delta_{i}\right)=P\left(Z_{pi}=1|\theta_{p},\alpha_{i},\gamma_{0i},\gamma_{1i}\right)\times f\left(X_{pi}|\theta_{p},\alpha_{i},\beta_{i},\delta_{i}\right)$$

$$k\left(X_{pi}|\theta_{p},\alpha_{i},\gamma_{1i}\right)=P\left(Z_{pi}=2|\theta_{p},\alpha_{i},\gamma_{1i}\right)$$

where $f\left(\theta_{p},\alpha_{i},\beta_{i},\delta_{i}\right)$ corresponds to the model-specific density function with support on the open interval (0, 1), $\beta_{i}\in R$ is an item location parameter, and $\delta_{i}\in R^{+}$ is an item dispersion parameter. So, in total, each model estimates five parameters per item. Note that the probability distribution of $X_{pi}$ is a mixture of a discrete distribution on {0, 1} and a continuous distribution on the open interval (0, 1). All three models are structurally the same except for the fact that, the probability density function, $f\left(\theta_{p},\alpha_{i},\beta_{i},\delta_{i}\right)$, is replaced with the corresponding model-specific function in the above equations. The specific density functions for the Beta, Simplex, and Samejima’s Continuous IRT models can be found in Appendix A.

**Figure 1:**
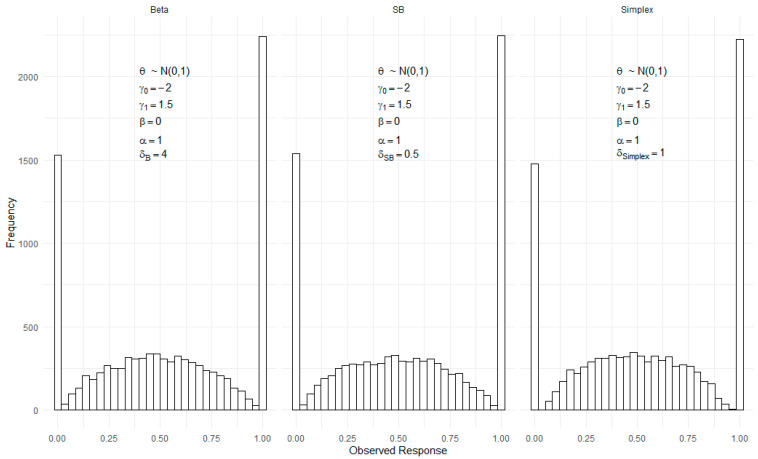
Comparison of model-generated response distributions for the Beta, SB, and Simplex IRT models. Latent proficiency is assumed to follow a standard normal distribution. All item parameters except the dispersion parameter were the same across models.

Figure 1 also visually compares the model-generated response distributions for a population with latent proficiency distributed as a standard normal distribution. This figure includes the three models (Beta, Simplex, and SB IRT models) for hypothetical items with identical item parameters ($\gamma_{0}$, $\gamma_{1}$, α, and β), but they differ in their dispersion parameters (δ) since dispersion parameters have different scales across the models. Despite the distinct mathematical formulations behind the density functions, the figure reveals significant similarity in the generated response distributions. This resemblance underscores the models’ robustness and adaptability to different types of distributions, particularly those that tend to cluster at the scale boundaries. For a comprehensive understanding of these models and their specific density functions, readers are encouraged to consult Molenaar et al. (2022) paper, where technical discussions are thoroughly presented.

### 2.3. Incorporating Collateral Information

Incorporating supplementary data about persons and/or items into IRT models offers numerous benefits. Prior studies have reported that including auxiliary information within the IRT framework can enhance both convergence and the precision of parameter estimation (Adams et al. 1997; de la Torre 2003; Hall 2007; Joo et al. 2022; Mislevy and Sheehan 1989; Tao 2009). Therefore, we also consider extending the models proposed by Molenaar et al. (2022) by incorporating information from two auxiliary variables related to person proficiency: writing and speaking scores. The models above can be extended by proposing a linear regression model of $\theta_{p}$ on the auxiliary variables,

$$\theta_{p}=\xi_{1}W_{p}+\xi_{2}S_{p}+\epsilon_{p}$$

where $W_{p}$ and $S_{p}$ are the observed writing and speaking scores for the pth examinee, $\xi_{1}$ and $\xi_{2}$ are the associated regression coefficients and $\epsilon_{p}$ is the error term. Both writing and speaking scores were standardized, so they have a mean of zero and unit variance before model fitting.

### 2.4. Model Fitting in Stan

*Prior Specifications.* We fit each model using the Stan software (Stan Development Team 2022c). The parameters of each model were estimated by implementing the No-U-Turn Sampler (NUTS) extension of the Hamiltonian Monte Carlo (HMC) algorithm, as implemented in the rstan and cmdstanr packages (Stan Development Team 2022a, 2022b) in R (R Core Team 2022). HMC is more effective for examining the posterior parameter space than conventional Markov Chain Monte Carlo (MCMC) algorithms, especially when dealing with intricate, high-dimensional models. Although traditional MCMC sampling algorithms can investigate the full target distribution for complex, high-dimensional models given sufficient time, approximating the posterior parameter distribution usually takes longer. HMC leverages auxiliary momentum variables, allowing each random draw to cover more ground in the parameter space, resulting in a quicker exploration of the entire target distribution. McElreath (2017) offers an accessible introduction to HMC with helpful visual aids, and more in-depth technical introductions can be found in works by Hoffman and Gelman (2011) and Betancourt (2018).

Similar to Molenaar et al. (2022), the following priors for the item location, item discrimination, and category thresholds were adopted

$$\beta ∼N\left(0,10\right)$$

$$log\left(\alpha \right)∼N\left(0,1\right)$$

$$\gamma_{0}∼N\left(0,10\right)$$

$$\gamma_{1}∼N\left(0,10\right),\;\gamma_{0}<\gamma_{1}$$

For the dispersion parameters,

$$\delta ∼N\left(0,10\right)$$

was used for the Beta IRT model while

$$log\left(\delta \right)∼N\left(0,1\right)$$

was used for the SB-IRT and Simplext IRT models. The prior for the error term in the regression model was specified as

$$\epsilon ∼N\left(0,\sigma^{2}\right)$$

$$\sigma^{2}=1-\left(\xi_{1}^{2}+\xi_{2}^{2}+2\xi_{1}\xi_{2}r\right),\;\sigma >0$$

where *r* is the observed correlation between writing and speaking scores. As mentioned, the observed writing and speaking scores were standardized with a mean of zero and unit variance before model fitting. Therefore, this specification implies a standard normal distribution as a prior for the marginal distribution of the latent person parameters. We also fit the models without the latent regression approach for comparison purposes. When no latent regression exists, the latent person parameters are directly modeled with a standard normal distribution.

*Parameter Estimation*. Parameter estimation was conducted using an out-of-sample prediction approach through cross-validation (Stenhaug and Domingue 2022). The complete dataset was divided randomly into six folds, ensuring each fold included at least one response from every participant with at least six responses (98.9% of respondents). The responses from participants with fewer than six responses were also randomly assigned to one of the six folds. Each model was fitted six times, with one fold excluded in each iteration. At the end, each model was also fitted to the entire dataset. This procedure allowed for comparing models based on their out-of-sample predictive performance. To achieve this, a model was fitted using a combination of five folds, and the estimated parameters were then used to predict observations in the excluded sixth fold. Four chains with random starting values were used when fitting the models, and each chain had 1000 iterations. The first 250 iterations in each chain were used as a warm-up, and the remaining 750 were used for inference. The convergence diagnostic measured by $\hat{R}$, modified by Brooks and Gelman (1998), was used to assess the convergence of every parameter.

### 2.5. Disclosure of the Use of AI or AI-Assisted Technologies

In the preparation and revision of this manuscript, the first author, Cengiz Zopluoglu, employed AI-assisted technologies to enhance the readability and linguistic quality of the text. Specifically, he utilized Grammarly for real-time grammar, spelling, punctuation, and clarity enhancements across the entire manuscript. Additionally, he consulted ChatGPT for specific feedback on cohesion, syntax, vocabulary, and grammar improvements, applying its suggestions to refine his writing. This approach was taken to ensure the manuscript meets the high standards of linguistic quality expected by the academic community. The final version of the entire manuscript after revisions was read and approved by both authors before finalizing the submission.

## 3. Results

### 3.1. Model Comparison and Prediction Error

Our approach to comparing models relies on assessing the effectiveness of person and item parameter estimates in predicting out-of-sample observations. We randomly divided the entire dataset into six folds, $X^{\left(r\right)}$ for *r* = 1, 2, …, 6. Following this, we fitted the models to each training set, excluding the rth fold $X^{\left(-r\right)}$. The derived person and item parameter estimates were then used to predict the out-of-sample observations in the excluded rth fold, using the relevant model equations. Subsequently, we computed the sum of squared errors for each rth fold,

$$SSE\left(X_{pi}^{\left(r\right)},{\hat{X}}_{pi}^{\left(r\right)}|\theta_{p}^{\left(-r\right)},\alpha_{i}^{\left(-r\right)},\beta_{i}^{\left(-r\right)},\delta_{i}^{\left(-r\right)},\gamma_{0i}^{\left(-r\right)},\gamma_{1i}^{\left(-r\right)}\right)=\underset{p}{\sum }\underset{i}{\sum }{\left(X_{pi}^{\left(r\right)}-{\hat{X}}_{pi}^{\left(r\right)}\right)}^{2}$$

We performed these calculations at each sampling iteration to estimate the posterior distribution of SSE, utilizing the parameter estimates obtained. To facilitate a more intuitive understanding of these results, we established a baseline SSE. We achieved this by predicting the value of each observation using the corresponding sample average.

**Figure 2:**
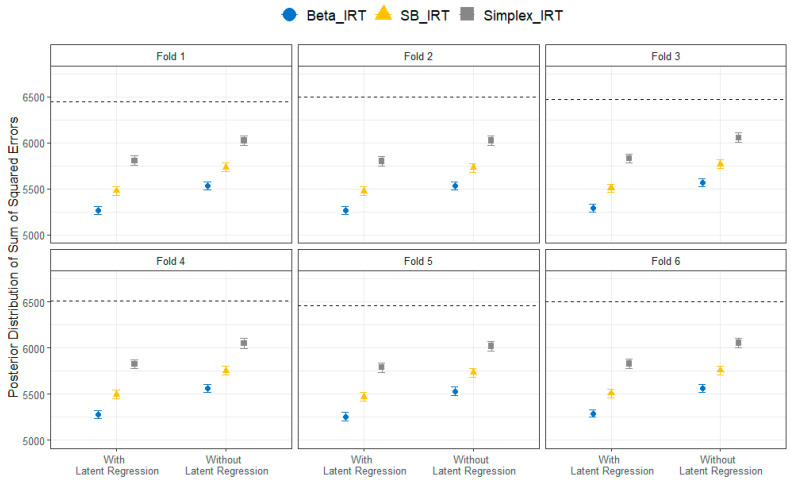
Comparison of the sum of the squared error of predictions across six folds for the Beta, SB, and Simplex IRT models with and without latent regression. The horizontal line for each fold represents the baseline prediction error when an average response is used. A smaller sum of squared error indicates better performance.

Figure 2 illustrates the out-of-sample prediction error for each fold for the Beta, SB, and Simplex IRT models with the collateral information included via latent regression and without collateral information during the estimation process. The outcomes were highly consistent across all six folds, with two key patterns emerging. Primarily, models that integrated collateral information from external variables via latent regression exhibited a significantly smaller prediction error, as measured by the sum of squared error, than those that did not use this collateral information. When not considering the collateral information, the Beta, SB, and Simplex IRT models displayed a proportional reduction in prediction error by 14.4%, 11.4%, and 6.8%, respectively, compared to the baseline SSE (represented by the horizontal lines in the figure). When the collateral information was included, the proportional error reduction improved to 18.6% for Beta IRT, 15.3% for SB-IRT, and 10.3% for Simplex IRT. Consequently, including collateral information contributed to an additional reduction in the prediction error by approximately 4% across models. The second crucial pattern that emerged was the superior predictive performance of the Beta IRT model across all folds regarding unseen responses. This superiority indicates that the Beta IRT model could be a strong candidate for future utilization in processing data from a similar assessment.

### 3.2. Model Fit

We evaluated certain aspects of the model fit by using the posterior predictive model-checking approach (PPMC; Rubin 1984). Essentially, PPMC contrasts actual data with model-predicted or generated data, utilizing various metrics that pinpoint areas where the model might not align well. If there is a noticeable divergence between the real data and the model’s predictions, it suggests that the model is not adequately capturing certain data facets. For this approach, we first generated data following the models by using the draw of person and item parameters from their respective posterior distributions provided by Stan. Visual representations are often the most user-friendly means to conduct these posterior predictive assessments. So, we created several visualizations to check the alignment between real data and model predictions. Given the superior predictive fit exhibited by models incorporating latent regression, we will present the model fit only for those models with latent regression.

**Figure 3:**
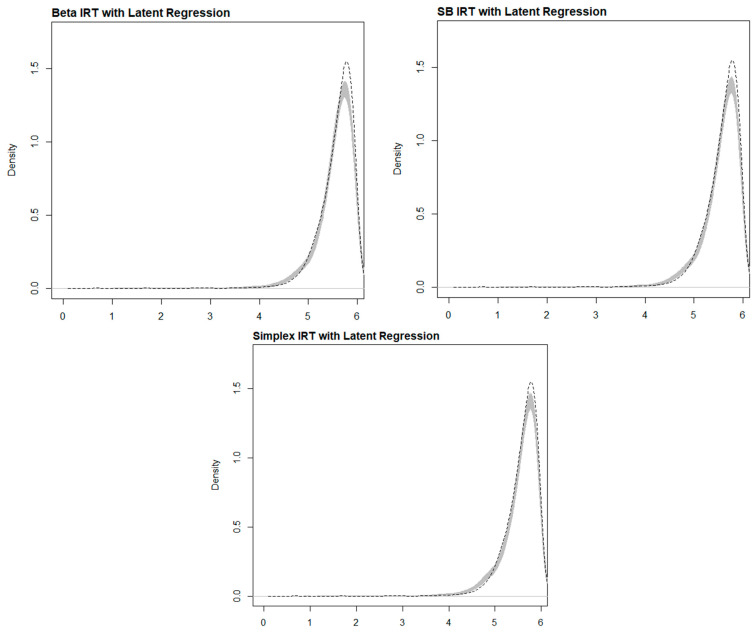
Density plots of observed sum score distribution (dashed line) and distributions of sum scores from 3000 posterior samples (gray area) for each model.

Table 1 and Figure 3 compare each model’s observed and posterior predictive score distributions, specifically for test takers who received six items. Given the continuous nature of scores, which can range from 0 to 6, both the table and figure illustrate that all three models—Beta IRT, SB IRT, and Simplex IRT—exhibit very similar performance characteristics. They commendably predict the observed score distribution, albeit with a noted reduction in skewness and kurtosis. However, the data seem to be heavier tailed than the model’s predictions, indicating possible limitations in the parametric models employed. Such models are, in essence, an approximation of any real data-generating model (DGM), and discrepancies tend to become more pronounced in higher moments, which amplify differences in the underlying distributions.

Furthermore, even if the parametric model accurately represented the majority of the data, real data are often subject to contamination from outliers due to idiosyncratic events. For instance, a test taker might become distracted and make an atypical error that would not replicate under different measurement conditions for the same person or item combination. This is particularly pertinent given that the test takers were required to type their responses for the items in this dataset; hence, even if they have completely understood the stimulus, there is still a potential for error during the input phase. Such outliers have a pronounced impact on higher moments, as they are probably more sensitive to this kind of data contamination.

**Figure 4:**
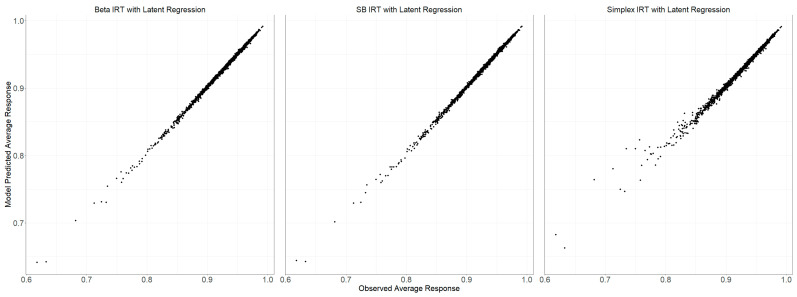
Comparison of average item scores from observed data and posterior predictive distributions of model-generated data.

Figure 4 illustrates the comparison between the average scores of actual observed data and those generated by the posterior predictive distributions of the models. This calculation is performed for each item, and Figure 4 summarizes across items. All three models—Beta, SB, and Simplex IRT—closely matched the observed average scores. The mean observed response for all items stood at 0.9307. In contrast, the averages for the model-predicted responses for the Beta, SB, and Simplex IRT models were 0.9315, 0.9318, and 0.9320, respectively.

**Figure 5:**
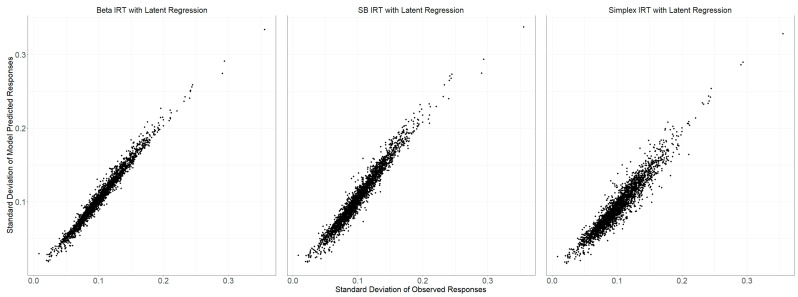
Comparison of standard deviations of item scores from observed data and posterior predictive distributions of model-generated data.

Figure 5 similarly presents a comparison across items between the standard deviations of observed item scores and those generated by the posterior predictive distributions of the models. The observed responses had an average standard deviation of 0.1021 across all items. On the other hand, the model-predicted responses slightly higher average standard deviations: 0.1053 (Beta), 0.1067 (SB), and 0.1110 (Simplex IRT). This data also highlights the Beta IRT model’s superior prediction accuracy for unseen data discussed earlier compared to the other two models. While all models were relatively consistent in reproducing average item scores, the Beta-IRT produced predictions with the smallest variance.

### 3.3. Parameter Estimates

Given the superior predictive fit exhibited by models incorporating latent regression, we will summarize the findings regarding parameter estimates specifically for these models. Each item in these models has five parameters, resulting in many item parameters per model. We evaluated the convergence of these item parameters, utilizing R-hat values. The majority of item parameters demonstrated high-quality convergence. Specifically, the R-hat values for 97.5%, 100%, and 99.9% of all item parameters for the Beta, SB, and Simplex IRT models were below the threshold of 1.05, indicating good convergence. We also examined the convergence for all person parameter estimates for each model and observed a similar quality. The R-hat values for 99.4%, 100%, and 99.9% of all person parameters for the Beta, SB, and Simplex IRT models were below the threshold of 1.05.

**Figure 6:**
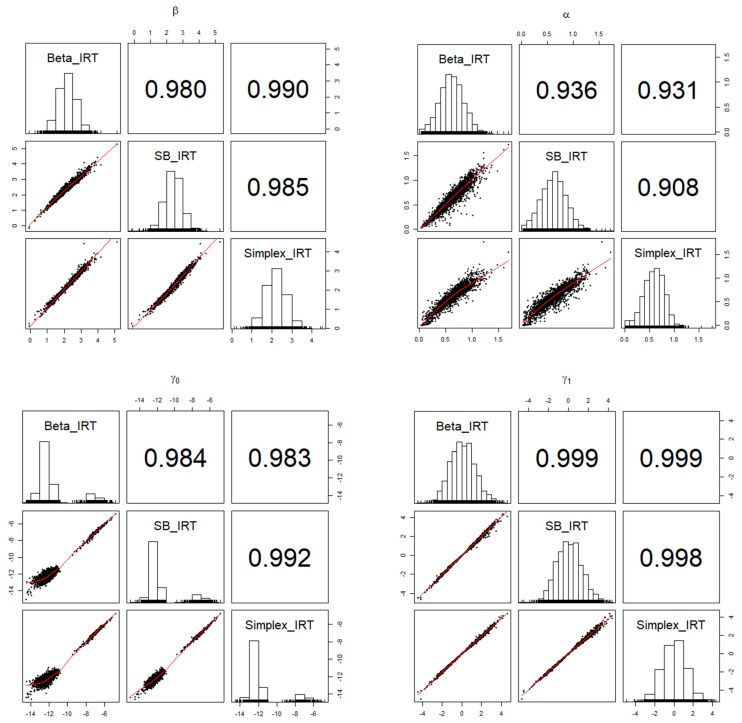
The relationships among the item parameter estimates obtained from Beta, SB, and Simplex IRT models.

**Figure 7:**
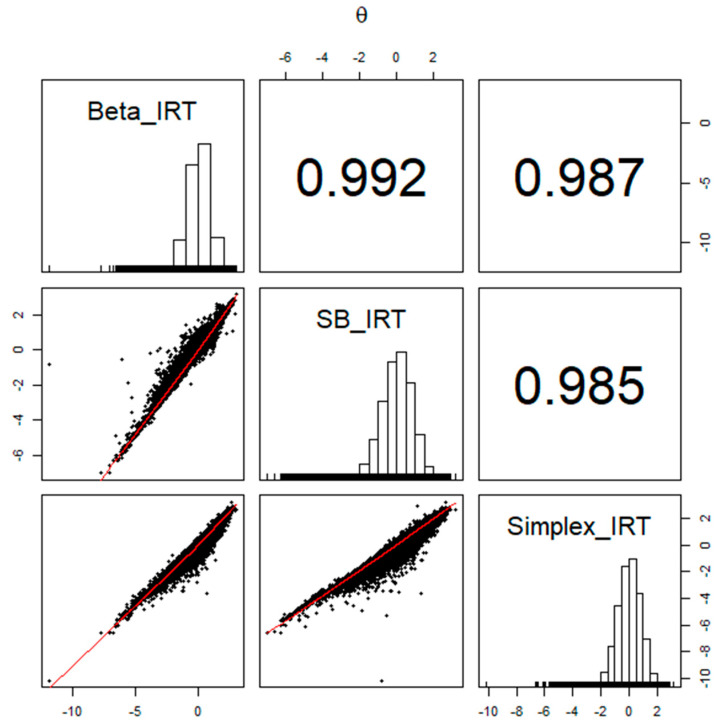
The relationships among the person parameter estimates obtained from Beta, SB, and Simplex IRT models.

Across the models, the item and person parameters exhibited strong similarities. The descriptive statistics for these model parameter estimates are presented in Table 2 and Table 3. Figure 6 and Figure 7 illustrate the relationships and correlations between item and person parameter estimates among the Beta, SB, and Simplex IRT models. Except for the α parameter, the parameter estimates from different models aligned very closely, with correlation values ranging from 0.980 to 0.999. However, the correlations were relatively lower for the α parameter, falling within a range of 0.91 to 0.94. The supplemental writing and speaking scores were significant predictors of the latent person parameters, and the estimated parameters were similar across models.

## 4. Discussion

The present study evaluated the performance of novel IRT models, specifically zero-and-one-inflated extensions of the Beta IRT, Simplex IRT, and Samejima’s Continuous IRT models, modeling grades of dictation tasks in a high-stakes English language proficiency assessment. We adopted a “predictive fit” approach, discussed and advocated by Stenhaug and Domingue (2022), to compare the models through cross-validation. Each independent fold of the whole response dataset included a randomly selected subset of responses from all test takers. The goal of the comparison was to measure how well a model predicts a person’s missing response in a certain fold using the item and person parameters estimated from the remaining folds.

Our findings underscore the potential of these models, especially when they incorporate collateral information, to provide accurate estimates and predictions. One of the most salient takeaways from our research is the superior predictive performance of the Beta IRT model regarding out-of-sample responses. This finding aligns with Molenaar et al. (2022), who found the Beta IRT model best fitting for 11 out of 22 scales they included in their study. We can argue that, among the models evaluated, the Beta IRT model holds the most promise for future applications in a similar assessment context, particularly when the task is administered adaptively. However, it is crucial to note that while the Beta IRT model demonstrated superior predictive accuracy for unseen data, the differences in model fit and parameter estimates among the models were not substantial. It is also important to consider the concept of fitting propensity when evaluating these models. While our study highlights the effectiveness of these models, especially the Beta IRT model, in high-stakes English assessments, a detailed comparison of their relative parsimony and flexibility remains an area for future exploration. Understanding whether the Beta IRT’s success is due to its greater flexibility than the other models could be crucial for its application. Future studies could focus on a fitting propensity analysis, like the work by Bonifay and Cai (2017) and Ergin (2020), to comprehensively evaluate these models. 

Our research has also highlighted specific gaps in the current understanding of novel IRT models studied in this paper. A notable challenge was the scarcity of established benchmarks or guidelines for evaluating model fit and item fit specifically for these advanced IRT models. This issue is particularly pressing given the fundamental importance of accurate model fit assessment in ensuring the validity and reliability of a model’s predictions. The absence of well-established evaluative criteria or methods leaves researchers and practitioners uncertain about the appropriateness of these newer models. In addressing this gap, our study primarily relied on heuristic visual checks based on model-generated data from respective posterior distributions of the item and person parameters. While established discrepancy measures for assessing model fit are relatively better researched and documented for traditional dichotomous and polytomous IRT models (e.g., Sinharay et al. 2006), such measures are not yet fully developed for these recent IRT models, particularly those for continuous responses or those introduced in recent research, such as by Molenaar et al. (2022). There is a need for dedicated research to develop and validate a comprehensive set of discrepancy measures for assessing model fit for these novel IRT models. Such research would enhance the credibility and utility of these models and equip researchers and practitioners with the essential tools they need to make informed decisions about model selection and interpretation. Considering the generally modest differences among the models in various performance metrics reported in our study, the selection in real-world settings could be based on alternative criteria such as interpretability, computational stability, and simplicity. 

As high-stakes computerized adaptive assessments for language proficiency continue to evolve, the integration of recent NLP technologies is anticipated to introduce novel item types. These advancements necessitate analytical models capable of accurately processing and interpreting data from such items. The models evaluated in our study, particularly their ability to handle bounded continuous outcomes, are well-suited for this emerging landscape. Our research indicates that these IRT models could be instrumental in managing the data complexities presented by NLP-driven assessment items, thereby supporting more nuanced and effective language proficiency evaluations.

## Figures and Tables

**Table 1:** Descriptive statistics for the sum scores from observed data and the average of posterior distribution of sum scores.

	Mean	SD	Skewness	Kurtosis
Beta IRT	5.56	0.36	−1.96	7.76
SB IRT	5.56	0.36	−1.87	6.72
Simplex IRT	5.56	0.37	−1.93	6.90
Observed Data	5.56	0.38	−2.90	22.63

**Table 2:** Descriptive statistics for item and person parameters estimated from the Beta, SB, and Simplex IRT models with latent regression.

	Beta IRT Model with Latent Regression				SB IRT Model with Latent Regression				Simplex IRT Model with Latent Regression			
Parameters	Mean	SD	Min	Max	Mean	SD	Min	Max	Mean	SD	Min	Max
θ	0.00	0.86	−11.86	3.05	0.00	0.85	−6.99	3.17	0.00	0.85	−10.17	3.20
β	2.18	0.53	−0.09	5.15	2.41	0.54	−0.16	5.27	2.20	0.51	0.12	4.52
α	0.61	0.23	0.02	1.69	0.62	0.25	0.03	1.72	0.59	0.22	0.00	1.77
$\gamma_{0}$	−11.62	1.97	−14.51	−4.77	−11.65	1.95	−15.05	−4.81	−11.64	1.96	−14.56	−4.77
$\gamma_{1}$	0.10	1.27	−4.47	4.62	0.09	1.28	−4.64	4.35	0.08	1.25	−4.93	4.38
*δ* *	3.53	0.97	−0.05	13.21	0.66	0.28	0.04	3.30	8.41	5.16	0.26	36.45

* The disturbance parameters (*δ*) are not comparable across the model.

**Table 3:** Estimated coefficients from the latent regression.

	$\mathrm{Writing}\;(\xi_{1})$		$\mathrm{Speaking}\;(\xi_{2})$	
	Posterior Mean	95% Credible Interval	Posterior Mean	95% Credible Interval
Beta IRT	0.351	(0.346, 0.354)	0.345	(0.340, 0.349)
SB IRT	0.347	(0.342, 0.351)	0.352	(0.347, 0.356)
Simplex IRT	0.330	(0.325, 0.334)	0.346	(0.342, 0.351)

## Data Availability

You can access our GitHub repository at https://github.com/czopluoglu/Duolingo_paper. It contains code samples that showcase the analyses performed on a simulated dataset that mimics the structure of real data analyzed in the study.

## Appendix A. Model-Specific Probability Density Functions

**SB_IRT Model**

$$f\left(X_{pi}{|\theta }_{p},\alpha_{p},\beta_{p},\delta_{p}\right)=\frac{1}{\sqrt{2{\pi \delta }_{i}}}\frac{1}{X_{pi}\left(1-X_{pi}\right)}\exp\left(-\frac{{\left(log\left(\frac{X_{pi}}{1-X_{pi}}\right)-\alpha_{i}\theta_{p}-\beta_{i}\right)}^{2}}{2\delta_{i}}\right)$$

**Beta IRT Model**

$$f\left(X_{pi}{|\theta }_{p},\alpha_{p},\beta_{p},\delta_{p}\right)=\frac{\Gamma \left(a_{pi}+b_{pi}\right)}{\Gamma \left(a_{pi}\right)\Gamma \left(b_{pi}\right)}X_{pi}^{a_{pi}-1}{\left(1-X_{pi}\right)}^{b_{pi}-1}$$

where $\Gamma \left(.\right)$ is the gamma function defined by $\Gamma \left(d\right)=\int_{0}^{\infty }t^{d-1}e^{-t}dt$, and

$$a_{pi}=e^{\frac{\alpha_{i}\theta_{p}+\beta_{i}+\delta_{i}}{2}}$$

$$b_{pi}=e^{\frac{-\left(\alpha_{i}\theta_{p}+\beta_{i}\right)+\delta_{i}}{2}}$$

**Simplex IRT Model**

$$f\left(X_{pi}{|\theta }_{p}i,\alpha_{i},\beta_{i},\delta_{i}\right)=\frac{1}{\sqrt{2{\pi \delta }_{i}{\left[X_{pi}\left(1-X_{pi}\right)\right]}^{3}}}\exp\left(-\frac{{\left(X_{pi}-\mu_{pi}\right)}^{2}}{2\delta_{i}X_{pi}\left(1-X_{pi}\right)\mu_{pi}^{2}{\left(1-\mu_{pi}\right)}^{2}}\right)$$

$$\mu_{pi}=\frac{1}{1+e^{-\left(\alpha_{i}\theta_{p}+\beta_{i}\right)}}$$
